# PERSONAL GROWTH AFTER GRAY DIVORCE: A LONGITUDINAL VIEW ON THE DISSOLUTION OF LONG-TERM MARRIAGES

**DOI:** 10.1093/geroni/igz038.2300

**Published:** 2019-11-08

**Authors:** Claudia Recksiedler, Katharina Loter, Oliver Arránz Becker, Pasqualina Perrig-Chiello

**Affiliations:** 1 German Youth Institute, Munich, Germany; 2 University of Halle-Wittenberg, Halle, Germany; 3 University of Bern, Bern, Switzerland

## Abstract

Going through a divorce is one of the most stressful life events across the life course with far-reaching ripple effects for individuals’ physical, mental, and often financial well-being. For some individuals, however, coping with marital dissolution and adapting to new social roles may lead to considerable gains in their reorientation in life and stimulate their personal development. This is captured in the concept of personal growth, which has been studied primarily in the context of becoming widowed, and through a cross-sectional lens. In our study, we track trajectories of personal growth after ending a long-term marriage of at least 15 years. We focus on divorce among adults in their second half of life because rates of grey divorce have been on the rise since the 1980s, yet longitudinal research on personal growth after marital dissolution remains sparse. We further examine whether trajectories of personal growth vary by gender, SES, reasons of divorce (e.g., sexual infidelity or drifted apart), and social support. Data stem from a Swiss panel on intimate relationships and marital dissolution among long-term married individuals conducted between 2012 and 2016 (N = 530). Random-effects group-specific growth curve models yielded similar—and slightly decreasing—trajectories of personal growth for divorced women and men over time. However, highly significant gender differences indicated that women showed higher levels of personal growth over the whole observation period. Because gender differences seemed to be fueled by social capital rather than economic assets, we discuss these results through the lens of a linked-lives perspective.

